# Determining direct, indirect healthcare and social costs for diabetic retinopathy management: a systematic review

**DOI:** 10.1186/s12886-024-03665-6

**Published:** 2024-09-30

**Authors:** Mawdda Benhamza, Maznah Dahlui, Mas Ayu Said

**Affiliations:** 1 Department of Social and Preventive Medicine, Faculty of Medicine, Universiti Malaya, Kuala Lumpur, 50603 Malaysia; 2 National Centre for Disease Control, Tripoli, Libya; 3 Department of Research Development and Innovation, Universiti Malaya Medical Centre, Kuala Lumpur, 50603 Malaysia; 4 Department of Health Policy and Administration, Faculty of Public Health, Universitas Airlangga, Surabaya, Indonesia; 5 Centre for Epidemiology and Evidence-Based Practice, Department of Social and Preventive Medicine, Faculty of Medicine, Universiti Malaya, Kuala Lumpur, 50603 Malaysia

**Keywords:** Diabetes, Diabetic retinopathy, Direct medical cost, Indirect medical cost, And indirect nonmedical cost

## Abstract

**Introduction:**

Diabetic retinopathy (DR) is a rapidly growing global public health threat; it affects 1 in 3 people with diabetes and is still the leading cause of blindness among the working-age population. The management of diabetic retinopathy is becoming more advanced and effective but is highly expensive compared to other ocular diseases.

**Aim:**

To report direct medical, indirect medical, and nonmedical costs of diabetic retinopathy in developed and developing countries through a systematic review.

**Methods:**

Related articles published in the PubMed, Google Scholar, and EMBASE electronic databases from 1985 to 2022 were identified using the keywords direct medical and indirect medical and social costs of diabetic retinopathy. However, previous systematic reviews, abstracts, and case reports were excluded.

**Results:**

Thirteen articles were eligible for assessing the economic burden of diabetes management and its complications. Our analysis revealed that increasing prevalence and severity of diabetic retinopathy (DR) are associated with higher direct and indirect healthcare expenditures. The impact of DR on working-age adults, leading to irreversible blindness in advanced stages, underscores the urgent need for cost-effective prevention and management strategies.

**Discussion:**

This study systematically reviewed the direct medical, indirect medical, and nonmedical costs of DR in developed and developing countries. Our findings highlight the significant economic burden of DR, emphasizing the importance of implementing effective prevention and management measures to alleviate costs and enhance patient outcomes.

**Conclusion:**

The substantial financial burden of DR necessitates a re-evaluation of current screening and management programs. Revision of these programs is crucial to improve quality of care, reduce costs, and ultimately achieve Sustainable Development Goal 3, which aims to ensure good health and well-being for all.

**Supplementary Information:**

The online version contains supplementary material available at 10.1186/s12886-024-03665-6.

## Introduction

Diabetic retinopathy (DR) is rapidly growing as a global public health threat. It affects 1 in 3 people with diabetes [1] and is still the leading cause of blindness among the working-age population. At least 2.2 billion people worldwide have visual impairments. In at least 1 billion– or almost half– of these cases, vision impairment could have been prevented or has yet to be addressed, whereas 3 million suffer from diabetic retinopathy, according to the last report updated by the World Health Organization in 2106 [2]. DR is the third leading cause of blindness [3]. However, it can be prevented if appropriate management is introduced early, which could reduce the risk of vision loss by 60% [4].

DR is caused by microvascular changes that occur in the retina and cause haemorrhages. The severity of haemorrhages depends on the duration of diabetes, glycaemic status, hypertension, smoking, and a high lipid profile. All these risk factors cause damage to the retina’s vasculature status in terms of ischaemia or bleeding, which leads to a change in the normal function of the retina.

The management of DR is comprehensive and requires diabetologist and ophthalmic subspecialty services to treat diabetes and its complications. Diabetes prevention methods are simple, cheap, and effective. One of the most preventive methods is tight blood sugar control.

The economic burden associated with DR is enormous; it is a social, healthcare, and government burden. Total healthcare costs for DR in the United States were estimated to be US$ 490 million in 2004, and the average annual total cost per DR patient was about US$ 629 [5]. In Sweden, the annual average healthcare costs of DR, proliferative diabetic retinopathy (PDR), and diabetic macular edema (DMO) were (US$ 93.6, US$ 334.1 and US$ 280.8) respectively [6]. Healthcare costs for low- and middle-income countries are still undetermined due to a lack of recording data.

Moreover, there is a hidden direct medical cost for DR, which is mental health. Unfortunately, DR-related vision loss has a detrimental effect on a patient’s mental health and can cause depression and a loss of interest in life, both of which have not yet been measured.

Direct healthcare costs include screening, follow-up, investigations such as labs and images, and treatments (laser, intravitreal injection, and vitrectomy). Indirect healthcare costs include transportation and accommodations. Indirect nonhealthcare costs include income loss, productivity loss, caregivers, visual aid assistance, disability, and blindness allowance.

Given the sacrality of financial information, especially for low- and middle-income countries in the DR. Then, it motivates a researcher to pay more attention to the community, healthcare workers, and government regarding the magnitude of the problem. The study aims to report direct medical, indirect medical, and nonmedical costs of DR in high-, middle-and low-income countries through a systematic review.

## Methods and materials

A systematic review was conducted based on the Preferred Reporting Items for Systematic Reviews and Meta-Analyses (PRISMA) criteria [7].

### Research strategy

The PubMed, Google Scholar, EMBASE, Research Gate, SPRING, and BMJ electronic databases were searched from January 1985 until October 2022. The study identified the keywords direct medical, indirect medical, and nonmedical costs of diabetic retinopathy (Appendix 1), while Table 1 is identified the type of costs with subdivision items.

**Table 1:** Type of costs with subdivision items

Type of costs	Items
Direct medical cost or called (healthcare cost or insurance payers )	Cost of DR screening (check-up) Cost of DR follow-up Cost of DR diagnostic tools (OCT, fluorescein angiography, ultrasound) Cost of treatment (glasses, intravitreal injection, panretinal photocoagulation, pars plana vitrectomy) In addition, some studies add the cost of diabetes Cost of DM check-up Cost of DM investigations Cost of DM treatment Cost of DM complications
Indirect -medical cost (patient’s cost)	Cost of transportation Cost of accommodation Cost of a healthy diet
Indirect non-medical cost (Societal cost or government cost )	Cost of time loss Cost of productivity loss Cost of income loss Cost of caregivers Cost of guide dog Cost of disability (vision aids) Cost of blindness allowance

### Inclusion and exclusion criteria

Studies included in a systematic review were original, complete economic evaluations (i.e., direct medical costs, indirect medical costs, and non-medical costs) of DR management. Studies excluded were previously published systematic reviews, abstracts, case reports, poster presentations, letters, comments, and other articles, including cost-effectiveness or cost-utility of DR screening and treatment with non-invasive intervention (intravitreal injection or pan-retinal photocoagulation) and invasive intervention (Pars plana vitrectomy), comparing the cost of treatment, the cost of diabetes without DR included, and abstracts.

### Screening process

To eliminate duplications, all identified records from multiple databases were entered into the ENDNoteX9 software, as well as, additional records from relevant journals discovered through the hand-search. After removing the duplicates, three reviewers screened the records (MD, MA, and MB) in two stages to evaluate for eligibility and the final section. The title and abstract were screened in the first stage, and the full articles in the second stage. During each stage of evaluation, reviewers assessed the records individually and then collected them to an agreement. Before finalising the records for the next screening step, disagreements were addressed through discussion. Disagreements among the three reviewers were discussed and resolved by consensus.

### Quality assessment of the study

The quality assessment of the study was done using the CHEQUE (Criteria for Health Economic Quality Evaluation) [8]. The scoring assessment was divided into 12 domains (Final-24 items) with a total score of 100; each choice of the level corresponds to a different weight that, cumulatively, sums up to the final score: Yes = full credit (i.e., the assigned importance score is multiplied by 1.0), somewhat = half credit (i.e., the assigned importance score is multiplied by 0.5), and no = 0 credit. Table 2 shows the scoring matrix for quality assessment methods.

**Table 2:** Scoring matrix for quality assessment methods

References	Round importance score	Score weighting assessment (Yes, somewhat, no)	Final score
Pushkar & Tiwari, 2022 [9]	100	49	49
Orji et al., 2021 [10]	100	37	37
Toth et al., 2020 [11]	100	69	69
Sasongko et al., 2019 [12]	100	72	72
Heintz et al. 2010 [6]	100	42	42
Schmier et al., 2009 [13]	100	51	51
Lee et al., 2008 [14]	100	51	51
Happich et al., 2008 [15]	100	49	49
Phillips et al., 1994 [16]	100	47	47
Rein et al., 2006 [5]	100	47	47
Morsanutto et al., 2006 [17]	100	47	47
Schmitt et al., 2004 [18]	100	47	47
Brien et al., 2003 [19]	100	47	47

Yes = credit 1; somewhat = credit 0.5; No credit = 0

### Data extraction and synthesis

A quantitative meta-analysis to explore possible relationships between the severity of DR and the DR cost could not be used, given that several studies had different study participants, settings, and countries of origin from different DR risk burdens. Studies were also heterogeneous in many aspects, including the type of cost included in the study, the type of cost measurements, and the analysis. Thus, the forest plot couldn’t be done due to the heterogenecity of the study.

Therefore, when available, data was extracted for all studies that included the direct medical, indirect medical, and non-medical costs. Data extraction included the following information: name of the study, authors, year of publication, and country of the study, methodology, population, included costs, direct medical costs, direct non-medical costs or indirect (patients’) costs, indirect (societal) costs, perspective years of costs, main outcomes, and main results.

## Results

The literature search of the selected databases revealed 415 articles (233 PubMed, 88 Google Scholar, 41 EMBASE, 25 Research Gate, 20 Taylor & Francis, 7 Spring, and 1 Elsevier). A total of 13 articles were reviewed and analysed. It was divided into three groups: the first group estimated the cost of DR complications, the second group measured the cost of diabetes with DR management, and the third group calculated the cost of the visual disorders, including the DR.

### Study screening

**Fig. 1:**
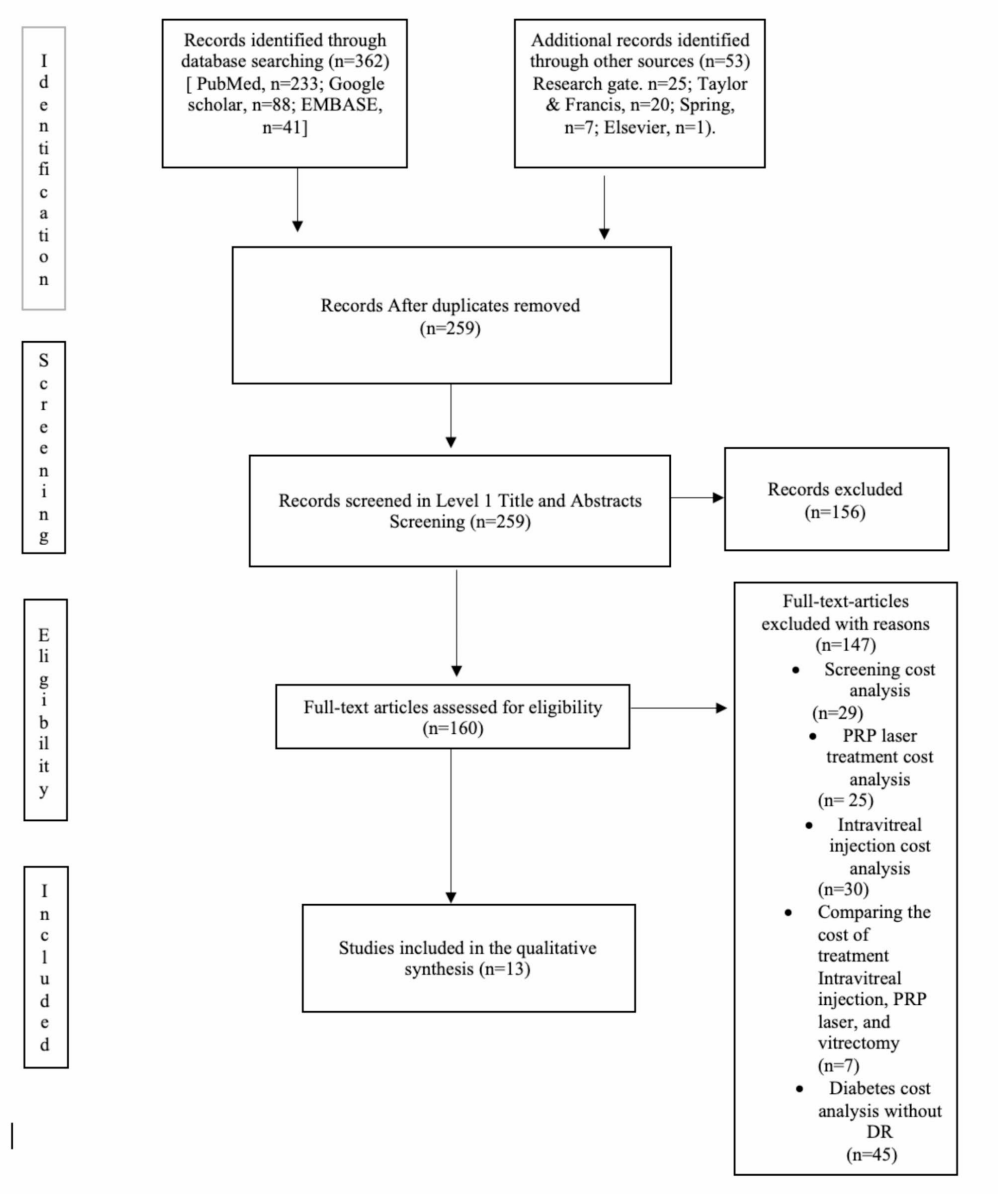
PRISMA flow diagram of the study selection process

Figure 1 shows a PRISMA flow diagram of the study selection process.

### Risk of bias

As indicated in Table 2, the risk of bias and quality of the included studies are rated between 37 and 72. Nine studies identified the study sampling, while three studies identified diabetes and its complications, including DR, and the last research identified the visual disorder, including DR. In addition, the studies classified the DR changes into mild, moderate, and severe NPDR, PDR, and maculopathy, or DMO, while other studies classified them into vision-threatening diabetic retinopathy (VTDR) and non-vision threatening diabetic retinopathy (non-VTDR). Lastly, 6 studies identified direct non-medical costs as indirect (patient’s) costs.

### Health, patient, and societal costs for DR

Thirteen studies were conducted in the cost analysis for direct medical, direct non-medical, or indirect medical (patients’), and indirect non-medical (societal) costs.

The characteristics of the systematic review were collected according to the study participants (DM with or without DR), study design, study setting at different healthcare levels (primary, secondary, tertiary, and community), type of healthcare system (private, government, and payer), and DR classification (no-DR, any DR, mild NPDR, moderate NPDR, severe NPDR, PDR, and maculopathy). Table 3 shows the characteristics of the studies included in the systematic review.

**Table 3:** Characteristics of studies included in the systematic review

Authors, year	Country	Study participants	Methodology	Healthcare setting/ data	Type of Healthcare system			DR Classification			
					Gov	Private	Payers	No-DR	mild, moderate, & severe NPDR	PDR	DMO
Pushkar & Tiwari, 2022 [9]	India	119 DR, previously Dx	Cross-section; 2016 to 2018	Community clinic / EMR	-	-	Yes/ sometimes medical institute	Yes	Yes	Yes	Yes
Orji et al., 2021 [10]	India	1000DR	Cross-section; Jan to Jun 2019	Tertiary eye care/EMR	-	-	Yes	Yes	Yes	Yes	Yes
Toth et al., 2020 [11]	Hungary	DM with or without DR	Cost of illness model; combing RAAB + DRM; in 2017- in 2045	Hospital/ EMR	Yes	-	-	Yes	Yes	Yes	Yes
Sasongko et al., 2019 [12]	Indonesia	DM with or with out DR	Cost of illness model; in 2016- in 2025	Hospital/ EMR	Yes	-	-	Yes	Yes	Yes	Yes
Heintz et al.,2010 [6]	Sweden	251,386 DM with or without DR	Cross-section; 2000 to 2007	CDWO/ and NDR	Yes	-	-	Yes	Yes	Yes	Yes
Schmier et al., [13] 2009	USA	DM with or without DR	Case-control; 1999 to 2004	Medicare insurance	Yes	-	-	Yes	Yes	Yes	Yes
Lee et al., 2008 [14]	USA	750,000 DM employer	Case control; 1999 to 2004	Health insurance date	-	Yes	Yes	Yes	Yes	Yes	Yes
Happich et al., 2008 [15]	Germany	207 DM with DR	Cross-section 2002	Hospital / EMR	Yes	-	-	-	Yes	Yes	Yes
Philips et al., 1994 [16]	Mexico	69 DM with or without DR	Cross-section; in 1985- in 1991	Large eye hospital/ MR	-	-	Yes	No	No	No	No
Rein et al., 2006 [5]	USA	Any visual disorder	Cross-section	Health insurance	Yes	Yes	-	No	No	No	No
Morsanutto et al., 2006 [17]	Italy	292 DM with or without DR	Cross-section; 2001 to 2002	Diabetic center/ EMR	Yes	-	-	No	No	No	No
Schmitt et al., 2004 [18]	Switzerland	1479 DM with or without DR	Cross-section; 1998 to 199	Primary care/EMR	Yes	-	-	No	No	No	No
Brien et al., 2003 [19]	Canada	DM	Cross-section ; 1994 to 1996	Ontario Case Cost Project; physician, Lab fee, reports, and literature	Yes	-	-	Yes	Yes	Yes	

CDWO (Care Data Warehouse in Ostergotland), NDR (National Diabetic Register), MR (Medical Record), DM (Diabetes Mellitus)

The systematic review included direct medical, direct non-medical or indirect (patients’), and indirect non-medical (societal) costs. Table 4 shows the direct and indirect costs included in the systematic review.

**Table 4:** Direct and indirect costs in the systematic review

Author, Year of Publication, Country	Included costs	Direct medical costs	Direct non-medical or indirect (patients’) Costs	Indirect (societal) costs	Perspective year(s) of cost	Main outcomes	Main results
Pushkar & Tiwari, 2022 India [9]	Direct medical and Indirect costs	Spectacles; PRP laser; Surgical procedure; Medicine; Clinical fee; Investigation.	Travelling; Diet control; Health classes and Miscellaneous.	Not- included	Payers in 2018	Cost	Average annual direct costs INR 1,901,000 (US$ 24,142.7); indirect costs INR 10,096,000 (US$ 128,219.2). Currency Exchange Rate INR 1 = US$ 0.0127
Orji et al., 2021, India [10]	Direct medical and non- medical costs	Consultation, Investigation; PRP laser; IV injection; and Surgery	Transportation; boarding; and loading	Not-included	Payers, medical institutes, societal and third party in 2019.	Cost	Total cost of 1000 pts INR 23,767. 838 (US$ 320,865.8). Median cost per patient INR 8,214 (US$ 110). Currency Exchange Rate 1 INR = US$ 0.0135
Toth et al., 2020, Hungary [11]	Direct medical and indirect costs	Screening mobile camera; General eye exam; OCT, FA & U/S; PRP laser; and Vitrectomy.	Loss of workdays	Not-included	Satutory health insuranc and patients in 2016 and in 2045	Cost	In 2016, screening cost with no DR per pt $3.0; mild/ observable DR $46.0; referable NPDR $207.8; proliferative DR $2376.2; proliferative DMO $3517.3. In 2045, screening with no DR per pt $12.8; mild/ observable DR $7.0; referable NPDR $2.7; proliferative DR $115.4; and proliferative DMO $69.
Sasongko et al., 2019 Indonesia [12]	Direct medical and indirect costs	Screening Mobil unit transport per pt; Camera maintenance; Photographer; Medications; nurse/ field assistant. Hospital costs includes Registration; General eye exam and referral letter per visit. Additional examination OCT per eye; PRP laser; IV injection; and Vitrectomy.	Transportation cost for screening per session and referral letter per session.	Loss of workdays per day and hospital treatment.	Healthcare perspective and patients perspectives in 2017 and in 2025.	Cost	In 2017, Screening costs without DR $20 million; mild to moderate NPDR $5.9 million; VTDR requiring laser PRP $2.6 million; VTDR requiring additional IVJ anti-VEGF $1751.7 million; advanced PDR needing vitrectomy $251.6 million. In 2025, screening cost mild to moderate NPDR $92 million; laser treatment for VTDR $901.9 million; additional IVJ anti-VEGF $6279 million; and vitrectomy for advanced PDR $1587 million.
Heintz et al., 2010, Sweden [6]	Direct medical costs	Cost of hospital overheads, ophthalmologist fees, eye examinations including photographs of the retina, PRP, hospitalizations, and other resource use related to vitrectomy	Not-included	Not-included	National cost per patient principle, in 2008	Cost	Total annual healthcare cost € 9.9 million US$ 10.494 million; representing an overall healthcare cost of € 106,000 million US$ 112.36 million per 100,000 population. Currency Exchange Rate EUR 1 = US$ 1.37
Schmier et al., 2009, USA [13]	Direct medical costs	Inpatient care per beneficiary in cohort cases (NPDR and PDR ) and control in cohort. Compared to outpatient care cases (NPDR and PDR ) and control. Inpatient care for beneficiaries with one or more claim cases (NPDR and PDR) and control. compared to outpatient care cases (NPDR and PDR) and control	Not-included	Not-included	Medicare claim service from 1999 to 2004	Cost	An average annual direct cost Inpatient care for control and case groups US$ 1,223 Outpatient care for control and case groups US$ 28.
Lee et al., 2008, USA [14]	Direct medical and Indirect non-medical costs	Medical services; mean hospital inpatient stays; emergency visits; outpatient visits; and other services. Mean of prescription drugs includes OHG and insulin	Not-included	Absenteeism and disablity	Payers, Medical and prescription claims and disability claim) for 12 months	Cost	Mean annual total direct & indirect costs of DR employees ($18218 vs. $3548), respectively. Direct and indirect costs of no-DR employees ($11898 vs. $2374), respectively.
Happich & Reitberger, 2008, Germany [15]	Direct medical cost (Statutory Health Insurance GKV) and indirect (social perspective) costs	Range of medical devices; Temporary working disability; and Other services; Hospitalization; Ophthalmologist fee; Medication; Other physician fee; Transport; Further non-drug therapy; and rehabilitation	Not-included	Range of medical devices; Temporary work disability; Other services; Early retirement; Hospitalization; Ophthalmic fee; Medication; Other physician fee; Further non-drug therapy; Home help services; Rehabilitation and nursing services; and transport	GKV and societal, in 2002	Cost	Average annual social perspective € 3.51 bn (US$ 3.3345 bn) while GKV perspective € 2.23 bn (US$ 2.1185 bn). Currency Exchange Rate EUR 1 = US$ 0.95
Rein et al., 2006, USA [5]	Direct medical, direct non-medical, and indirect costs	Outpatient and inpatient visits.	Long term care; nursing home; guide dogs; independent living services for elderly blind individuals; national library services for the blind; physically handicap; and American printing house.	Productivity loss, decrease wages.	Medicare claims and MarketScan claims in 2004	Cost	Total cost of Major visual disorders $35.4 bn; Direct medical cost US$ 16.2 bn. Other direct costs US$ 11.1 bn. Productivity loss US$ 8 bn.
Phillips et al., 1994, Mexico [16]	Direct medical direct non-medical and indirect costs	Checkup; angiogram; echography; internal medicine; laboratory; PRP; cryotherapy; vitrectomy; cataract; and eyeglasses.	Transportation accommodation, and others.	Time loss; productivity loss; income loss; Disability; and percentage poor sight 51%.	Payers in 1985 and in 1991	Cost	Average cost per pt to pt Mex$ 1,549,515 (US$ 80,574.78). Average social cost of hospital treatment per pt Mex$ 1,877,035 (US$ 97,605.8). Currency Exchange Rate Mex$3 = US$1
Morsanutto et al., 2006, Italy [17]	Direct medical costs	Drugs; Visit to specialist; visit to GP; diagnosis; hospitalizations	Not-included	Not-included	National Health Service in 2002	Cost	Mean total healthcare cost of DM € 1909.67 US$2,272.50. Mean cost of single DM-related complication € 1808.17 US$ 2,151.72. Total cost per pt with DR € 1,329.9 US$ 1,582.59. Currency Exchange Rate € 1 = US$ 1.19
Schmitt et al., 2004, Switzerland [18]	Direct medical costs	Drug costs; Ambulatory costs include consultations, Outpatient diagnostic procedures, outpatient invasive procedures, and laboratory tests. Hospital care.	Not-included	Not-included	Swiss Health Insurance in 1998.	Cost	Total direct cost CHF 0.582 US$ 0.874 bn; represented 2.2% total country healthcare. Currency Exchange Rate CHF 1 = US$ 1.50
O’Brien et al., 2003, Canada [19]	Direct medical costs	Outpatient visits, labs, and laser treatment.	Not-included	Not-included	Health Insurance and government, in 2000	Cost	Blindness cost in the state US$ 1794.35 Currency Exchange Rate Cand$1 = US$ 0.85

STDR ( Sight-threatening Diabetic Retinopathy); IVJ (Intravitreal Injection); FA (Fluorescein Angiography); OCT (Ocular Coherence Tomography);

RAAB-DR (Rapid Assessment of Avoidable Blindness with the Diabetic Retinopathy Module; VTDR (Vision- threatening Diabetic Retinopathy;

PRP (Pan-retinal photocoagulation); NPDR (Non-proliferative Diabetic Retinopathy); PDR (Proliferative Diabetic Retinopathy; DME (Diabetic

Macular Edema)Macular Edema). EMR (Electronic Medical Records); MR (Medical Records); bn (billion); pt (patient).CDWO (Care Date

Warehouse in Ostergotland); NDR (National Diabetic Register); GKV(Gesetzliche Krankenversicherung); and GP (General practitioner)

#### Direct medical (Healthcare) costs

In 2022, Pushkar and Tiwari estimated the average direct medical costs, which included the cost of spectacles INR 50,000 (US$ 635,00), laser INR 315,000 (US$ 4,000.5), surgical procedures INR 360,000 (US$ 4,572), medicines INR 360,000 (US$ 4,572), clinical fees INR 264,500 (US$ 3,359), investigations INR 549,500 (US$ 6,978.65) [9].

In 2021, Orji et al., the total costs of 1000 patients were INR 23,767. 838 (US$ 320,865), where the total direct cost for STDR (sight threat diabetic retinopathy) vs. non-STDR (non-sight threat diabetic retinopathy) was (INR 31,820; US$ 429.57 vs. INR 14,356; US$ 1938), respectively, while the cost of care for paying to non-paying was (INR 22,800; US$ 307.8 vs. 0.00 costs), respectively [10].

In 2020, Toth et al., reported the direct medical costs per patient in Hungary as follows: US$ 8.6 for screening via mobile camera, US$ 8.1 for a general eye exam, US$ 7.4 for an OCT exam (optical coherence tomography), US$ 9.2 for an FA exam (fluorescein angiography), US$ 5.9 for a U/S exam (ultrasound), US$ 7.6 for PRP laser (pan-retinal photocoagulation) per eye, US$ 1086.9 for IVJ (intravitreal injection), and US$ 858.6 for vitrectomy [11].

In 2019, Sasongko et al., estimated direct medical costs per patient in Indonesia, including screening, mobile unit transport at US$ 0.46, camera maintenance at US$ 0.31, photographer at US$ 0.15, medications at US$ 0.31, and nurse/field assistant at US$ 0.15. Hospital costs include registration, general eye examination, and referral letter per visit at US$ 28.1; additional examination OCT per eye at US$ 28.1; laser treatment at US$ 118.7; IVJ at US$ 330.1; and vitrectomy at US$ 1552.2 [12].

In 2010, Heintz et al., estimated the average annual direct medical costs in Sweden of 25,386 persons with diabetes with or without DR patients was any DR per patient € 72 (US$ 98.64); severity of DR such as BR; PDR; maculopathy; and combined PDR with maculopathy (€ 26/US$ 35.62; € 257/US$ 352.09; € 216/US$ 295.92 and € 433/US$ 593.21), respectively [6].

In 2009, Schmier et al., estimated the direct medical cost of DM with or without DR on patients 65 years of age and older in the United States. The cost was divided into outpatient and inpatient costs (NPDR and PDR) and control. The payment was divided into the average payment per beneficiary in the cohort and the average payment for beneficiaries with one or more claims. Inpatient care per beneficiary in cohort cases (NPDR and PDR) and control in cohort were (US$ 5, US$ 16, and US$ 3), respectively. Compared to outpatient care cases (NPDR and PDR), control was (US$ 292; US$ 1207; US$ 90), respectively. Inpatient care for beneficiaries with one or more claim cases (NPDR and PDR), control (US$ 4499; US$ 4217; US$ 5017), respectively, compared to outpatient care cases (NPDR and PDR), control (US$ 382; US$ 1285; US$ 231), respectively [13].

In 2008, Lee et al., estimated direct costs among DM employees with or without DR in the United States. Direct costs for DR and non-DR employees include mean annual hospital inpatient stays of US$ 1033, emergency visits of US$ 2, outpatient visits of US$ 2919, and other services of US$ 3376. The mean of the prescription drugs, including oral hypoglycemic drugs, insulin, and non-hypoglycemic drugs, was (US$ 145, US$ 241, and US$ 434), respectively. The cost differences were significant across DR employee subgroups: DME/non-DME (US$ 28 606/$16 363); PDR/non-PDR ($30 135; $13 445; *p* < 0.0001). DR with/without photocoagulation ($34 539; $16 041; *p* < 0.0001), and DR with/without vitrectomy ($63 933; $17 239; *p* < 0.0001) [14].

In 2006, Rein et al., estimated direct costs among patients complaining of visual disorders aged 40 and older in the United States. The direct medical costs for each condition were roughly 6.8 billion US dollars for cataracts, 5.5 billion US dollars for refractive error, 2.9 billion US dollars for glaucoma, 575 million US dollars for age-related macular degeneration (AMD), and 493 million US dollars for diabetic retinopathy in 2004. The outpatient costs of DR per patient were divided into physician and hospital costs (US$ 468; US$ 127), respectively, and the inpatient cost of DR per patient was US$ 0.00 [5].

In 2008, Happich et al., estimated the direct medical costs range of medical devices was US$ 325.85, temporary working disability US$ 188.1, other services US$ 11.4, hospitalization US$ 134.9, ophthalmologist fee US$ 121.6, medication US$ 23.75, additional physician fees US$ 22.8, transport US$ 13.3, further non-drug therapy US$ 16.15, and rehabilitation US$ 7.6 [15].

In 1994, Phillips et al., estimated direct and indirect costs among DM with or without DR. Results Include average checkup US$ 31.096, angiogram US$ 11,050, echography US$ 6,400, internal medicine US$ 2,000, labs US$ 20,000, laser US$ 17,980, cryotherapy US$ 14,000, vitrectomy US$ 63,866, cataract US$ 116,566.6, eyeglasses US$ 9,333.3 [16].

Three studies estimated the direct costs of diabetes and its complications. Morsanutto et al., in 2006 estimated the mean annual healthcare costs of 299 DM patients to be € 1909.67 (US$ 2,272.5073) per patient. The total cost per patient with DR was € 1329.91(US$ 1,582.59), which was divided into the cost of drugs € 819.36 (US$ 975.03), visit specialist € 123.10 (US$ 146.48), visit to GP (general practitioner) € 66.58 (US$ 79.23), diagnostics € 184.96 (US$ 220.10), and hospitalization € 135.91 (US$ 161.73) [17].

Schmitt et al., in 2004 estimated mean annual direct medical costs of € 2,323 (US$ 3,484.5) per year. Hospitalization costs € 1,856 (US$ 2,784), contributing 53% of total costs. Medication costs € 1,059 (US$ 1,588.5), contributing 30%. Ambulatory costs, including consultations, outpatient diagnostic and invasive procedures, and home care services by nurses, are € 1,181(US$ 1,771.5), contributing 17%. The cost of diabetic retinopathy complications per year per patient € 2,425 (US$ 3,637.5) [18].

O’Brien et al., in 2003 estimated the direct medical costs of diabetes and its complications in event costs were PDR, macular edema, and both US$ 379, US$ 423, and US$ 495, respectively. The state cost of US$ 40, comprised of additional monitoring by an ophthalmologist, is the same for both conditions [19].

#### Indirect medical (Patient) costs

In 2022, Pushkar and Tiwari, estimated the average indirect medical costs include the cost of traveling INR 125,500 (US$ 1,593.85), diet control INR 846,000 (US$ 10,744.2); health classes INR 971,500 (US$ 12,338.05); the miscellaneous cost INR 7,254,000 (US$ 92,125.8) [9].

In 2021, Orji et al., estimated indirect medical costs of 1000 patients, including transportation by bus/ or train within Hyderabad was INR 74 (US$ 0.99), < 200 (KM) from Hyderabad was INR 518 (US$ 6.99), 200–500 KM was INR 1095 (US$ 14.78), 500–800 KM was INR 1465 (US$ 19.77), 800–1300 KM was INR 2738 (US$ 36.96), 1300–2000 KM was INR 3108 (US$ 41.95), > 2000 KM was INR 3922 (US$ 52.94). However, transportation by airplane was 200–500 KM from Hyderabad was INR 6731 (US$ 90.86), 500–800 Km was INR 4958 (US$ 66.93), 800–1300 KM was INR 5402 (US$ 72.92), 1300 − 200 Km was INR 5846 (US$ 78.92), and > 2000 Km was INR 9768 (US$ 131.86). For the accommodation 500–800 KM was INR 1406 (US$ 18.91), 800–1300 Km was INR 1406 (US$ 18.91), 1300–2000 KM was INR 1406 (US$ 18.91), and > 2000 KM INR 1406 (US$ 18.91) [10].

In 2020, Toth et al., estimated the indirect medical costs in Hungary of lost workdays at US$ 29.8 per day [11].

In 2019, Sasongko et al., estimated indirect medical costs per patient in Indonesia, including patients’ transportation cost for screening per session, were US$ 1.92, and referral letter per session was US$ 7.69 [12].

In 2006, Rein et al., estimated indirect medical costs among patients complaining of visual disorders aged 40 and older in the United States. Direct non-medical costs include long-term nursing homes (US$ 10.96) billions, guide dogs (US$ 0.062) billions, independent living services for older and blind individuals (US$ 0.029) billions, national library services for the blind and physically handicapped American Printing House for the Blind (US$ 0.016) billions [5].

Phillips et al. In 1994, estimated indirect medical costs among DM with or without DR. Direct patient costs include transportation of US$ 20,500, accommodation of US$ 11,666.6, and others of US$ 4,166.6 [16].

#### Indirect non-medical (Societal) costs

In 2019, Sasongko et al., estimated indirect costs per patient in Indonesia, including hospital treatment per visit, were US$ 7.69, and loss of workdays per day was US$ 6.15 [12].

In 2008, Lee et al., estimated indirect costs among DM employees with or without DR including absenteeism (US$ 422) and disability (US$ 752) [14].

In 2008, Happich et al., estimated the indirect costs including a range of medical devices, US$ 325.85; temporary work disability, US$ 311.6; other services, US$ 182.4; early retirement, US$ 173.76, hospitalization US$ 134.9; ophthalmic fee US$ 121, medication US$ 27.05; additional physician, fees US$ 22.8, further non-drug therapy US$ 16.15, home help services US$ 16.15, rehabilitation US$ 7.6, nursing services US$ 3.8 and transport US$ 19.95 [15].

In 2006, Rein et al., estimated indirect costs of visual disorders aged 40 and older in the United States, including productivity loss, decreased workforce participation (US$ 6.3) billions, and decreased wages (US$ 1.73) billions [5].

In 1914, Phillips et al., estimated indirect costs among DM with or without DR in Mexico, including time loss, an average number of effective days lost 2 days, productivity loss of US$ 11,333.3, income loss, an average value of income loss per visit of US$ 5,666.6, and disability, a percentage of poor sight 51% [16].

Overall, estimating Purchasing Power Parity (PPP) proves challenging due to the variations in costs from country to country, even when factoring in currency exchange rates. This divergence is attributed to several factors, including differences in healthcare systems, such as the availability of healthcare facilities and health insurance. Additionally, variations in government economies, such as a social security system, income levels, and support for blindness allowance and visual aids, contribute to the observed differences.

## Discussion

Diabetic retinopathy (DR) and its complications impose significant burdens on the community, healthcare system, and government levels. The management of DR is characterized by its complexity and necessitates both medical and nonmedical interventions, which causes the total expenses of DR to increase. According to the American Diabetes Association (ADA), the United States incurs an annual expenditure on diabetes in 2022 of 412.9 billion US dollars. This cost encompasses direct medical costs, amounting to 306.6 billion US dollars, and lost productivity costs, totalling 106.3 billion US dollars [20]. Approximately 30% of individuals with diabetes are affected by diabetic retinopathy. The number of diabetic retinopathy is predicted to reach 16 million by 2050, and diabetes-related vision loss is expected to cost 500 million US dollars annually [21].

A systematic review evaluated the direct and indirect medical and non-medical costs among patients with DR. The studies reported that the expense of DR increased in parallel with the severity of the disease. On the other hand, the heterogeneity of study designs and outcome measures made it difficult to compare the total costs of direct and indirect costs, which influenced the drawing of conclusions. Additionally, the limited number of studies on estimating direct and indirect costs presented an additional obstacle to gathering sufficient information regarding healthcare and economic status.

Two studies in India explained the substantial economic burden associated with DR, in addition, the payment system methods in India were related to the medical institute model, with the absence of medical insurance or a third-party payment system, which may demonstrate the poor compliance because the institute model, which represented the out-of-pocket spending. Orji et al. in 2021, increased the cost of STDR compared to non-STDR because of the need for vitreoretinal surgery in STDR compared to cataract surgery in non-STDR. The cost-benefit analysis of treatment identified a threefold difference in the average medical cost per eye for blind patients compared to those treated with good vision (INR 26,270; US$ 355 vs. INR 8,510 and US$ 115), respectively [10]. Notably, only one-third of the patients were females, which may potentially be attributed to societal discrimination [22].

The primary cause for the escalation in healthcare expenditures in Hungary and Indonesia was the administration of anti-VEGF injections and vitrectomies, which accounted for 86.7% of the total healthcare cost of DR both in 2016 and in 2045 in Hungary. It accounted for 71% and 18% of the total healthcare cost of DR for intravitreal injection and vitrectomy in Indonesia for 2017 and 2025, respectively. Hungary’s cost-per-patient value in 2016 was also lower than Indonesia’s in 2017 (US$450.8). However, this difference is misleading due to the significantly higher prevalence of DR in individuals with DM in Indonesia (43.15%) compared to Hungary (20.1%). In addition, a resident ophthalmologist’s gross monthly basic salary immediately after graduation was US$ 905.8 in Hungary and US$ 5094.1 in Germany, which explains the low healthcare cost in Hungary [11, 12].

In Sweden, the expenses of any DR were PDR € 257 (US$ 352.09), maculopathy € 216 (US$ 295.92), and both complications combined € 433 (US$ 593.21) reported [6]. In contrast to the findings of a German study and a Canadian study, the estimations from the German study give an average cost of € 468 (US$ 444.6) (for patients with PDR and € 681 (US$ 646.95) for macular edema [15]. In the Canadian study, the combined costs for PDR, macular edema, and complications were estimated to be € 284 (US$ 241.4), € 254 (US$ 215.9) and € 333 (US$ 283.05), respectively [19].

The United States conducted three primary investigations to assess the direct and indirect costs. In 2009, Schmier et al. examined the yearly Medicare expenditures for individuals with diabetes, both with and without diabetic retinopathy. There were substantial increases in Medicare payments for beneficiaries with PDR and a moderate increase among beneficiaries with NPDR compared to controls. Nevertheless, it appears that the study may have overestimated its findings because the frequency of diabetic retinopathy complications was more profound in younger patients when compared to older patients [13].

Lee et al. in 2008, found that DR employees had significantly higher costs than other employees with diabetes but without DR, with a mean annual difference mean annual comorbidity-adjusted cost difference equal to US$ 2032. Substantial cost differences also existed within DR subgroups. Employees with PDR cost more than twice as much on average as NPDR employees, and employees with DME had mean annual costs that were approximately 75% higher than non-DME employees. The cost differences identified in this study highlight the extent to which DR imposes a substantial economic burden on employers over and above the cost of diabetes. In addition, the estimation of indirect costs did not include loss of productivity due to presenteeism (i.e., when a worker is present but not fully functioning on the job due to a medical condition), mortality, and costs [14].

Rein et al. in 2006, conducted a study to assess the substantial economic impact that major visual disorders, such as DR, impose on society. It estimated that the overall financial burden of these visual disorders among individuals aged 40 years or older in the United States amounts to 35.4 billion US dollars. This includes 16.2 billion US dollars in direct medical expenses, 11.1 billion US dollars in other direct costs, and 8 billion US dollars in productivity losses [5].

Phillips et al. in 1994, claimed that the substantial costs associated with diabetes could be reduced by hospitals offering financial aid and providing other methods such as reducing visits, waiting time, long demand for treatment, and promoting patient and relative complications associated with DM [16].

Three studies have conducted estimations on the direct medical expenses associated with diabetes and its complications. In a study conducted by Morsanutto et al. in 2006, it was revealed that diabetic patients with single or multiple complications incurred an average cost of € 1673.79 (US$ 1,948.97) and € 2666.69 (US$ 3,173.36), respectively. In contrast, diabetic patients without complications had an average cost of € 911.74 (US$ 1,084.97) [17]. Schmitt et al. in 2004, found that the overall direct cost of DM amounted to € 0.582 (US$ 0.87) billion, which accounted for 2.2% of the total healthcare costs in the country. It also highlights the substantial burden imposed by the costs of DM and its related complications on the country [18]. In 2003, O’Brien et al. reported that Canada had the highest prevalence of DM and that its complications were significant healthcare issues, affecting nearly 6% of the Canadian population [19]. It has been widely acknowledged that a considerable portion of the public healthcare budget is allocated to healthcare expenditures associated with the complications of diabetes [23]. Furthermore, a study estimated the cost of blindness attributable to diabetes to be Can$ 2,111 (US$ 1,794.35) [19].

In 2024, a study was to describe the costs associated with DR and to evaluate its economic impact in Jorden. The DR-associated cost was significantly higher with insulin-based regimens, longer duration of DM, higher HbA1c levels, and worse stage of DR at presentation was associated with higher DR-related costs ( for high risk of PDR US$ 4,218.579 and low risk of PDR US$ 2,840.022 versus for NPDR US$ 2,031.2 and for no DR US$ 701.616), for the presence of DMR at the presentation was associated with higher DR-related cost (for both eyes DME US$ 3,846.903 versus one eye DME US$ 3,299.259). In addition, increased the sessions of intravitreal injection, increased sessions of laser, and surgical operations [24].

The economic burdens of DR-related direct and indirect costs are linked directly with the severity of DR and indirectly with the duration of DM, treatment regimen, and the level of HbA1c. To mitigate the burden associated with DM, such as DR, we should focus on DM management. In addition, to prevent the DR complication from progressing or getting worse, we should focus on screening tools rather than treatment plans.

## Limitation

The study does not include a cost-effectiveness or cost-utility analysis; there were no inflation cost measures except in Canada, then inflating the Canadian value to a 2000 Canadian dollar. Not all studies calculate direct and indirect costs, and heterogeneity makes it difficult to pursue meta-analysis.

## Conclusion

Studies have found that the financial burden associated with the management of DR is exorbitant, encompassing both direct and indirect costs. All studies concluded that direct and indirect DR costs are considerable and challenging to control, particularly as the disease severity worsens.

To mitigate the diabetic retinopathy burden, we should focus on preventive methods like regular eye screening, control of blood sugar, and control of risk factors that will delay the progression into advanced DR changes.

The difference between direct and indirect costs is tremendous; the cost charge from country to country is different, which has to play another role for the DR management.

## Electronic supplementary material

Below is the link to the electronic supplementary material.

Supplementary Material 1

## Data Availability

The database used and/ or analyzed during the current study is available from the corresponding author upon reasonable request.
