# Supplementary material for: Determining direct, indirect healthcare and social costs for diabetic retinopathy management: a systematic review
Source: BMC Ophthalmol. 2024 Sep 30;24:424. doi: 10.1186/s12886-024-03665-6 (PMC11441148; doi:10.1186/s12886-024-03665-6)
Supplement: Supplementary file 1 — Supplementary Material 1 [file 12886_2024_3665_MOESM1_ESM.docx]

**Appendix 1** Search Strategy and the MeSH terms and keywords

| Databases | Duration | Population | MeSH terms and Keywords |
| --- | --- | --- | --- |
| Electronic databases (PubMed, GoogleScholar, EMBASE, Research Gate, SPRING, BMJ) | From 1^st^ January 1985 until 31^st^ October 2022 | Adults’ diabetic patients with or without DR | Direct medical, indirect medical or non-medical costs of DR |
